# Randomized Trials Built on Sand: Examples from COPD, Hormone Therapy, and Cancer

**DOI:** 10.5041/RMMJ.10082

**Published:** 2012-07-31

**Authors:** Samy Suissa

**Affiliations:** Center for Clinical Epidemiology, Lady Davis Institute, Jewish General Hospital, and Departments of Epidemiology and Biostatistics, and of Medicine, McGill University, Montreal, Canada

**Keywords:** Cohort studies, drug effectiveness, drug indications, observational studies, randomized controlled trials, scientific evidence

## Abstract

The randomized controlled trial is the fundamental study design to evaluate the effectiveness of medications and receive regulatory approval. Observational studies, on the other hand, are essential to address post-marketing drug safety issues but have also been used to uncover new indications or new benefits for already marketed drugs.

Hormone replacement therapy (HRT) for instance, effective for menopausal symptoms, was reported in several observational studies during the 1980s and 1990s to also significantly reduce the incidence of coronary heart disease. This claim was refuted in 2002 by the large-scale Women’s Health Initiative randomized trial. An example of a new indication for an old drug is that of metformin, an anti-diabetic medication, which is being hailed as a potential anti-cancer agent, primarily on the basis of several recent observational studies that reported impressive reductions in cancer incidence and mortality with its use. These observational studies have now sparked the conduct of large-scale randomized controlled trials currently ongoing in cancer.

We show in this paper that the spectacular effects on new indications or new outcomes reported in many observational studies in chronic obstructive pulmonary disease (COPD), HRT, and cancer are the result of time-related biases, such as immortal time bias, that tend to seriously exaggerate the benefits of a drug and that eventually disappear with the proper statistical analysis.

In all, while observational studies are central to assess the effects of drugs, their proper design and analysis are essential to avoid bias. The scientific evidence on the potential beneficial effects in new indications of existing drugs will need to be more carefully assessed before embarking on long and expensive unsubstantiated trials.

## INTRODUCTION

The randomized controlled trial is the fundamental paradigm to evaluate the effectiveness of medications in the clinical setting. It is the essential study design required by regulatory agencies to approve the marketing of a drug or a new indication for an existing drug. Despite extensive pre-approval trials, medications can have important unintended side-effects even if used properly. The epidemiological approach of observational studies has been recognized as an essential tool to address post-marketing drug safety issues and study the actual effects of medications as used in the population, a different situation from the experimental setting in which the drugs were developed and approved. This approach is particularly important for less frequent but severe adverse events or long-term adverse effects that cannot and could not be detected by the randomized trials required for initial drug approval. Moreover, the use of existing computerized databases arising from the routine collection of data in the usual care of patients has become essential for the rapid conduct of these observational studies in this field called pharmacoepidemiology. For example, health care databases worldwide have been used to rapidly assess the risks and benefits of several drugs such as NSAIDs, beta-agonists, anti-depressants, anti-hypertensives, statins, gastric-acid suppressants, corticosteroids, and many others, on major disease outcomes.^1^–^9^

Another less common situation where observational studies have been used is to uncover new indications for drugs that are already on the market or to assess the effectiveness of such available drugs in the same indication but on new outcomes not studied in pre-approval trials. An example of the effectiveness of a drug on new outcomes is that of hormone replacement therapy (HRT), an effective treatment for menopausal symptoms. After widespread use, several observational studies reported in the 1980s and 1990s that HRT may also significantly reduce the incidence of coronary heart disease. An example of a new indication for an old drug is that of inhaled corticosteroids in chronic obstructive pulmonary disease (COPD) and metformin, an anti-diabetic medication, which has been receiving much attention recently as a potential anti-cancer agent, primarily on the basis of several observational studies that reported impressive reductions in the incidence of and mortality from cancer. These observational studies formed the impetus for the conduct of major large-scale randomized trials.

In this paper, we show that the spectacular effects reported in many of the observational studies that have been conducted in this context are the result of time-related biases, particularly immortal time bias which tends to exaggerate the benefits observed with a drug. We also show how the studies could have avoided this bias, and the ones that did actually reported null effects. With this knowledge, it is unlikely that randomized trials would have been conducted.

## INHALED CORTICOSTEROIDS IN COPD

Chronic obstructive pulmonary disease, a disease that encompasses emphysema, chronic obstructive bronchitis, and small airway obstruction, is characterized by largely irreversible airflow obstruction.^10^ It currently affects around 10% of the population over the age of 40 years and has recently become the third leading cause of death in the US.^11^,^12^ The pharmacological treatment of COPD has generally consisted of bronchodilators. However, because of the presence of inflammation in COPD, inhaled corticosteroids, which had been shown to be highly effective for the treatment of asthma, were readily adopted in COPD in the 1980s despite the fact that no randomized controlled trials had yet evaluated their effectiveness in this indication.

The earliest randomized controlled trials to evaluate inhaled corticosteroids in the treatment of COPD were only published in the late 1990s. The first seven trials found no improvement in the decline of lung function over time and, except for the last two trials, found no reduction in exacerbation rates with various inhaled corticosteroids (ICS) compared with placebo, over periods ranging from 6 months to 3 years.^13^–^19^ In the early 2000s, the next wave of randomized controlled trials all involved the evaluation of inhaled corticosteroids combined with a long-acting beta-agonist.^20^–^25^ Most of these trials reported significant effects on lung function and reductions in exacerbation rates with the combination therapy, while the effects of inhaled corticosteroids alone were equivocal. Thus, the totality of these trials can be concluded to imply that any effectiveness of these medications is driven primarily by the long-acting beta-agonist component.^26^ Despite this weak evidence or even evidence to the contrary, inhaled corticosteroids are prescribed to over 70% of COPD patients in the United States and Europe.^26^

During this same period, several observational studies of large population-based cohorts, conducted using health care databases, were published. These studies, using a simplistic time-fixed definition of exposure, reported highly spectacular reductions in all-cause mortality of 30% to 40% with ICS use, alone or in combination with a long-acting beta(2)-agonist (LABA).^27^–^30^ By using a time-fixed definition that does not allow drug exposure to vary over time, these studies introduced a bias known as “immortal time bias” that we describe in this observational study context.^31^–^35^

### Observational Study 1

To describe the role of immortal time bias in these studies, we use the first of these published studies.^27^ This study used a cohort design to assess whether the use of inhaled corticosteroids after discharge from hospital for COPD was effective at reducing the risk of COPD readmission or all-cause death. All 22,620 patients over 65 years of age admitted to hospital for COPD in Ontario, Canada, between April 1992 and March 1997 were identified from this Province’s health insurance database. The patients were followed from the date of discharge for up to 1 year, or earlier if they were readmitted or died, in which case follow-up ceased at those points. The 11,481 patients who filled at least one prescription for an inhaled corticosteroid during the first 90 days after discharge were classified as users. The remaining 11,139 who did not were classified as non-users. An intent-to-treat analysis was performed on the basis of this classification using a proportional hazards regression model, accounting for several covariates. The resulting adjusted hazard ratio of all-cause death was found to be 0.71 (95% CI 0.65–0.78) for inhaled corticosteroid use relative to non-use, a 29% reduction.

**Figure 1:**
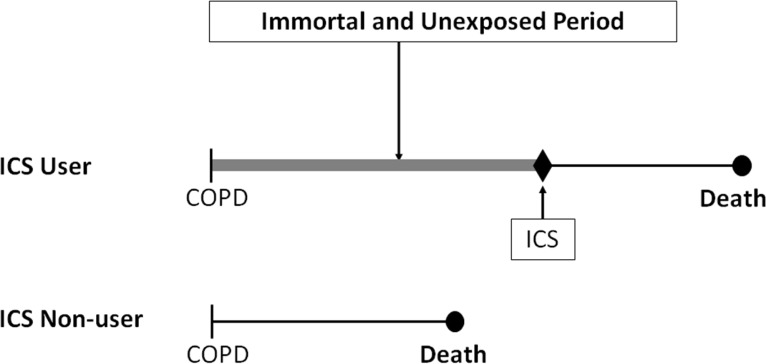
**Illustration of immortal time bias in the Sin and Tu observational cohort study of inhaled corticosteroids in patients discharged with COPD.^27^** The top patient received a prescription of ICS within 90 days after discharge and is classified as an ICS user for the entire follow-up time. The time between cohort entry and the first ICS prescription is thus immortal (thick line), since the subject must survive to receive this first ICS prescription, and is also misclassified as exposed to ICS when in fact unexposed, leading to immortal time bias.

*Immortal time bias* is introduced in this study by the definition of exposure in the cohort analysis. In this cohort study, a subject is considered exposed when an inhaled corticosteroid is dispensed at any time during the 90-day period after discharge. Hence, to be exposed, a patient must first survive the time until they receive that first prescription in that 90-day period. Thus, the time span between the date of discharge and the date of the first prescription of inhaled corticosteroids is called “immortal” because no deaths can occur during this period (Figure 1). More important, however, is the fact that subjects are classified as “users” of the drug during this immortal period even though the patient was not exposed until the first prescription was dispensed in that 90-day period. The misclassification of this time period as “exposed” when in fact it should have been classified as unexposed will engender immortal time bias. The solution is simply to use a time-dependent approach to data analysis that permits the patient to be classified as unexposed from cohort entry until the date of their first prescription, after which they can be classified as exposed. Methods based on person-time using Poisson models or more sophisticated techniques such as the Cox proportional hazards models with time-dependent exposure are available to account correctly for this problem.

To illustrate the principle behind this bias, we used the simple person-time approach (estimating rate ratios with Poisson models to compute confidence intervals) on the data provided in the paper, after rounding the numbers for simplicity and making assumptions for unreported data. Thus, we considered that there were 12,000 patients per group, with a mean follow-up of 9 months, so that each group generated 9,000 person-years of follow-up, with 2,400 deaths occurring during follow-up, 1,000 in the ICS user group and 1,400 in the non-users. For the sake of illustration, we simply assumed that the mean delay between cohort entry (discharge) and the first ICS prescription among the ICS users was at 45 days, i.e. midway into the 90-day period used to define exposure. Table 1 shows that this would result in 1,500 immortal person-years of no ICS exposure misclassified as ICS exposed. The resulting rates of death for ICS users (1,000/9,000 = 11.1 per 100 person-years) and for non-users (1,400/9,000 = 15.6 per 100 person-years), based on these misclassified immortal person-years, produce a crude rate ratio of 0.71 (95% CI 0.66–0.77), which suggests a significant reduction in mortality. However, by properly reclassifying these 1,500 immortal person-years as unexposed, the rates would become 1,000/(9,000–1,500) = 13.3 per 100 person-years for ICS use and 1,400/(9,000+1,500) = 13.3 per 100 person-years for non-use, resulting in a corrected crude rate ratio of 1.0 (95% CI 0.92–1.08), suggesting no benefit whatsoever.

**Table 1:** Comparison between biased time-fixed data analysis and corrected time-dependent data analysis for the cohort study of inhaled corticosteroid (ICS) use and all-cause mortality in chronic obstructive pulmonary disease (COPD).^27^

	**ICS Users (*n* = 12,000)**			**ICS Non-Users (*n* = 12,000)**			**Crude Rate Ratio (95% CI)**
	
	**Deaths**	**Person-Years**	**Rate per 100 Person-Years**	**Deaths**	**Person-Years**	**Rate per 100 Person-Years**	

**Biased Time-Fixed Analysis**							
Immortal and unexposed person-time	0	1,500*		0	0		
At-risk person-time	1,000	7,500		1,400	9,000		
**Total**	1,000	9,000	11.1	1,400	9,000	15.6	0.71 (0.66–0.77)

**Corrected Time-Dependent Analysis**							
Immortal and unexposed person-time	0	0		0	1,500*		
At-risk person-time	1,000	7,500		1,400	9,000		
**Total**	1,000	7,500	13.3	1,400	10,500	13.3	1.00 (0.92–1.08)

* Bold values represent the person-years of cohort follow-up misclassified as ICS exposed in the time-fixed analysis and properly reclassified as unexposed in the time-dependent analysis.

To illustrate further this bias with actual data from another cohort, we replicated the study using data from the computerized health care databases of Saskatchewan, Canada, to form the cohort of patients who were hospitalized for COPD between January 1, 1990 and December 31, 1997.^31^ The cohort included 979 subjects, of whom 389 subjects either died or were re-hospitalized for COPD during the 1-year follow-up. During the first 90 days of follow-up, 39% were dispensed an inhaled corticosteroid. Using the same approach as Sin and Tu, namely the Cox proportional hazards models with time-fixed exposure, the hazard ratio was 0.69 (95% CI 0.55–0.86), suggesting a strong benefit with this drug. However, using the correct analysis with the Cox proportional hazards models with time-dependent exposure that properly classifies exposure as ICS non-use during the immortal time period and as ICS use only after the date of dispensing of the first ICS prescription, the hazard ratio becomes 1.00 (95% CI 0.79–1.26), suggesting no such benefit with ICS.

### Observational Study 2

A variation of this bias was seen in another observational study of inhaled corticosteroids (ICS) in the treatment of chronic obstructive pulmonary disease (COPD), which claimed in its title to present “results from two observational designs free of immortal time bias.”^29^ This claim turned out to be in fact erroneous and reflected a grave misunderstanding of immortal time bias. The authors identified, from the United Kingdom’s General Research Practice Database (GRPD), the cohort of all 4,398 patients aged 50 years and older hospitalized for COPD from 1990 to 1999. Cohort entry was taken as the date of discharge, with 1-year follow-up until readmission to hospital for COPD or death. Patients were considered exposed to ICS if they received a prescription of ICS on the same day of discharge. Using a propensity scores matched cohort analysis, the hazard ratio of COPD readmission or death associated with ICS use was 0.69 (95% CI 0.52–0.93), suggesting a significant 31% reduction in this outcome with ICS use.

**Figure 2:**
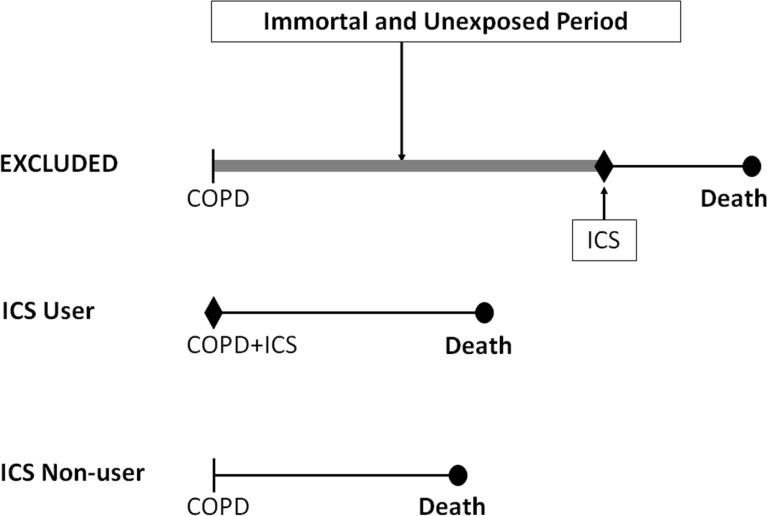
**Illustration of immortal time bias in the Kiri et al. observational cohort study of inhaled corticosteroids in patients discharged with COPD.^29^** The top patient received the first prescription of ICS during the year after discharge and was excluded. The time between cohort entry and the first ICS prescription is immortal and unexposed (thick line) and, with the subsequent exposed time, should have been included in the analysis to avoid immortal time bias.

*Immortal time bias* is in fact introduced again with the definition of ICS exposure. It is stated that “treatment status was defined on the same day of discharge,” so that all 1,091 patients who were prescribed ICS on the day of discharge were correctly classified as ICS-exposed. However, of the remaining 3,307 patients, the non-users of ICS were incorrectly taken as merely the 538 patients “who were never exposed to ICS in their entire (one-year) follow-up period.” To comply with their stated Methods, they should have used all 3,307 patients from the cohort who were not prescribed ICS *on the day of discharge*. By excluding the 2,769 patients who were not prescribed ICS on the day of discharge but received an ICS later in the year of follow-up, the authors excluded a crucial component of follow-up time which is both unexposed and immortal, thus introducing a significant degree of immortal time bias in the results (Figure 2). Had the authors followed the correct method they described in the paper, namely to use “only patients whose treatment status was defined on the day of discharge,” they would have included all 3,307 such patients in the non-ICS group, and the rate ratio of COPD hospitalization or all-cause death with ICS would have been 1.48, not the reported 0.70.^33^

### Observational Study 3

A further variation of this bias was seen in another observational study of ICS in COPD, also conducted using the GRPD. The cohort of 1,045 COPD patients treated with both an ICS (fluticasone) and a LABA (salmeterol) was compared with the cohort of 3,620 COPD patients who used other bronchodilators but not ICS or a LABA. The 3-year survival of the two cohorts was compared using survival analysis techniques. After adjusting for confounders, the combined users of ICS+LABA had a significant 52% lower mortality (hazard ratio 0.48; 95% CI 0.31–0.73), and the users of ICS only had a significant 38% lower mortality (hazard ratio 0.62; 95% CI 0.45–0.85) than the reference group of other bronchodilator users.

*Immortal time bias* is introduced in the hierarchical definition of exposure, where exposure is first assessed to identify the “exposed” cohort, namely those patients who received ICS+LABA. Only then was the “unexposed” reference group identified from the remaining patients as those who did not receive ICS or LABA, but only short-acting bronchodilators. However, many “exposed” subjects had used short-acting bronchodilators prior to their start of ICS+LABA, consistent with the stepped-care approach to COPD treatment. Thus, several subjects from the “exposed” group were in fact “unexposed” before switching to this exposure status. More importantly, however, this pre-exposure time during which subjects were “unexposed” is an immortal period since these subjects, in switching from the “unexposed” status to the “exposed” status, will necessarily do so alive. Had they died before switching, they would by definition have belonged to the unexposed group. Thus, the bias occurs because valid unexposed person-time of follow-up with no deaths is not accounted for in the reference rate of death. This results in an artificial increase in the rate of death of the reference group, leading to a spurious appearance of effectiveness. This bias was illustrated in another cohort of COPD patients, with the hazard ratio changing from a highly significant 0.66 (95% CI 0.57–0.76) to a non-significant 0.94 (95% CI 0.81–1.09) after properly accounting for this bias.^32^

### The TORCH Randomized Trial

In 2007, a large-scale randomized controlled trial was published, comparing an ICS+LABA (fluticasone+salmeterol) combination with placebo, LABA alone, or ICS alone, over a period of 3 years, on the primary outcome of death from any cause.^36^ Of the 6,112 randomized patients, all-cause mortality was 12.6% in the ICS+LABA combination group, 15.2% in the placebo group, 13.5% in the LABA group, and 16.0% in the ICS group. The hazard ratio of death for the ICS+LABA combination compared with placebo was 0.82 (95% CI 0.68–1.00), while compared with ICS alone it was 0.77 (95% CI 0.64–0.93). Moreover, for ICS alone compared with placebo, the hazard ratio was 1.06 (95% CI 0.89–1.27). The authors concluded that the mortality reduction with combination therapy did not reach the predetermined level of statistical significance.

As these results were inconclusive, a further analysis of the data as a 2×2 factorial design of ICS (yes/no) and LABA (yes/no) was performed to improve the power and tease out the independent contribution of each component of the combination.^37^,^38^ The interaction term to assess whether there is synergy between the two drugs was found to be non-significant (*P* = 0.32) suggesting that the combination of ICS and LABA is not particularly more effective than the two components added independently. Moreover, the factorial analysis showed that the LABA component is associated with a significant 17% reduction in mortality (RR 0.83; 95% CI 0.74–0.95; *P* = 0.0043), while the ICS component provides no reduction in mortality (RR 1.00; 95% CI 0.89–1.13; *P* = 0.99).^38^

In essence, all observational studies suggesting a reduction in mortality with ICS use were shown to be flawed with immortal time bias, and proper re-analyses to avoid this bias eliminated any apparent protective effect of ICS.^31^,^32^,^34^,^35^ In fact, Observational Study 2, described above, was specifically designed to emulate the TORCH randomized trial. It is now evident that the significant 38% and 52% potential reductions in mortality with ICS reported in this cohort study, in stark contrast with the absence of effects found in the TORCH randomized trial, were the result of immortal time bias.

## HRT AND CORONARY HEART DISEASE

Hormone replacement therapy (HRT) is an effective treatment for menopause, demonstrated to reduce menopausal symptoms, including hot flashes, vaginal dryness, and joint pain, to improve sleep quality, and to prevent bone loss and the related osteoporotic fractures. After their successful introduction, HRTs became the most commonly prescribed drugs in the United States, with the number of prescriptions increasing from 13.6 to 31.7 million between 1982 and 1992.^39^ This widespread use reflected not only their known beneficial effects, but also the newer postulated benefits of this therapy. Indeed, several observational studies conducted during this period reported major reductions in coronary heart disease (CHD) in women using HRT. In 1998, a meta-analysis of these multiple observational studies reported a summary relative risk for CHD of 0.70 (95% CI 0.65–0.75) with use of estrogen-only HRTs and 0.66 (95% CI 0.53–0.84) with use of estrogen-progestin combined HRTs.^40^

In 2002, the Women’s Health Initiative (WHI), a large-scale randomized controlled trial of postmenopausal women conducted to evaluate the benefits of combined estrogen and progestin compared with placebo in over 16,000 women with a uterus, reported its findings after 5 years of follow-up.^41^ With respect to cardiovascular outcomes, the study found hazard ratios of 1.29 (95% CI 1.02–1.63) for coronary heart disease, 1.41 (95% CI 1.07–1.85) for stroke, and 1.22 (95% CI 1.09–1.36) for total (arterial and venous) cardiovascular disease. Here again, as in the case of inhaled corticosteroids in COPD, many of the observational studies had major methodological flaws, including immortal time bias. We describe below some of these studies and their major source of bias.

**Figure 3:**
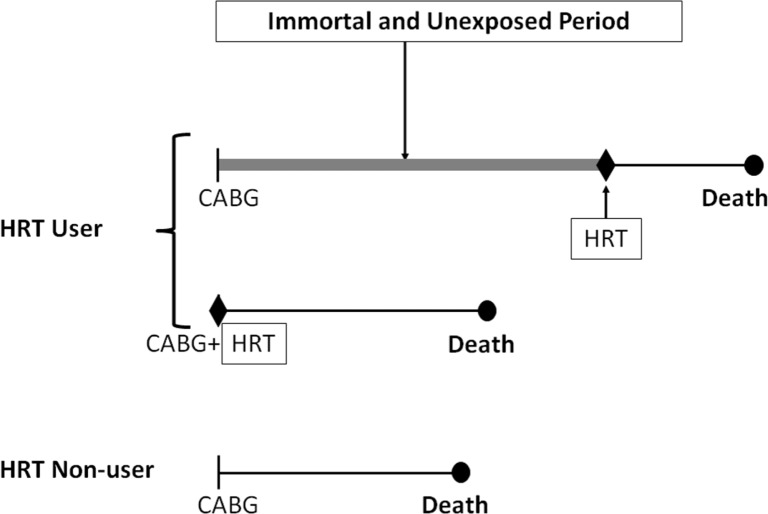
**Illustration of immortal time bias in the Sullivan et al. observational cohort study of HRT in patients undergoing CABG surgery.^42^** The top patient received her first prescription of HRT well into follow-up, long after CABG. The time between cohort entry and the first HRT prescription is immortal and unexposed (thick line) and should have been included in the analysis with the third patient, while the subsequent exposed period should have been included in the analysis with the second patient, to avoid immortal time bias.

The first is the cohort study assessing the effect of HRT on mortality after coronary artery bypass grafting that included 1,098 women undergoing coronary artery bypass graft (CABG) surgery in a single US medical center.^42^ The study population was selected from all women undergoing coronary arteriography at the Baptist Memorial Hospital, Memphis, Tennessee, between 1972 and 1989. Information on HRT use was obtained from the cardiac catheterization reports and from annual follow-up questionnaires sent to the patients’ physician, while the outcome of death was established by reports from the physician or family until 1991. Subjects were defined as HRT users if treated with estrogens at the time of admission for angiography or if estrogen was listed as a current medication on any response to the follow-up questionnaire. As a result, 92 were noted to have received HRT, including 42 at the time of CABG and 50 any time during follow-up. These were compared with 1,006 non-users who had not received HRT at baseline or any time during follow-up. Five- and ten-year survival was 98.8% and 81.4%, respectively, in the HRT users and 82.3% and 65.1% in the non-users. The Cox proportional hazards model resulted in a remarkable 62% reduction in mortality with HRT use (hazard ratio 0.38; *P* < 0.001). The authors concluded that HRT use after surgery significantly improves the survival of postmenopausal women with coronary artery disease. *Immortal time bias* was introduced in this study classifying the 50 women who initiated HRT sometime during follow-up as exposed to HRT during the entire follow-up (Figure 3). Thus a woman who had her CABG in 1972 and only initiated HRT use in 1982 was called exposed to HRT when in fact she was not exposed between 1972 and 1982. The fact that she started in 1982 implies she was alive on that date, introducing a 10-year “immortal” period misclassified as exposed to HRT instead of unexposed, which will necessarily generate immortal time bias in this study.

The second example is the Study of Osteoporotic Fractures, based on a prospective cohort identified from four US communities in Oregon, Minnesota, Maryland, and Pennsylvania.^43^ The cohort included 9,704 women 65 years or older, who were observed between 1986 and 1994. Of these, 14.1% reported use of HRT for at least 1 year. During an average follow-up of 6 years, 11.8% died, and after adjustment for confounders the all-cause mortality rate was 31% lower in users of HRT (RR 0.69; 95% CI 0.54–0.87). The RR was 0.95 (95% CI 0.68–1.32) among short-term users of HRT compared with 0.55 (95% CI 0.40–0.75) among long-term users. The authors concluded that HRT is associated with lower overall mortality rates. *Immortal time bias* was again introduced in this study by defining use of HRT, not exclusively at the baseline interview, but by updating this exposure information at the third clinical visit, i.e. 3.5 years after cohort entry. Here again, HRT exposure was misclassified as exposed during this 3.5-year immortal time period when the women were actually not yet users and only started use at that point. Another aspect of this bias is highlighted by the duration of use results reporting that women who used HRT for more than 10 years had a stunning 45% lower mortality than non-users. This analysis as performed by the authors is flawed since the 10 years of use guarantees that a woman is still alive after 10 years, while non-users can die soon after cohort entry. In contrast, the analysis of short-term use, which inherently has much less such guaranteed survival, found only a non-significant 5% lower mortality than non-users.

The third example is the NHANES study of HRT and stroke, which involved a cohort of 1,910 women entering the study between 1971 and 1975, with long follow-up until 1987.^44^ There were 250 cases of stroke that occurred during the average 12 years of follow-up. To assess the effects of HRT, the authors used the HRT data collected at the first wave of follow-up of this cohort, namely during the period 1982–1984. After adjustment, the rate of stroke was 31% lower in HRT users (RR 0.69; 95% CI 0.47–1.00), while stroke mortality was 63% lower (RR 0.37; 95% CI 0.14–0.92). The authors concluded that HRT use is associated with a decrease in risk of stroke incidence and mortality in white postmenopausal women. Here again, we note that *immortal time bias* is introduced in this study by defining use of HRT, not at the baseline questionnaire, but by around 10 years later, at the first wave of follow-up. The women who replied to the 10-year follow-up questionnaire to indicate that they used HRT were necessarily alive at that time and therefore contributed a guaranteed survival of 10 years to the analysis.

Finally, the fourth example involves a cohort of 2,436 women undergoing elective percutaneous transluminal coronary angioplasty (PTCA) between 1982 and 1994.^45^ Of these, the 137 postmenopausal women receiving HRT were matched with 200 postmenopausal women not receiving HRT and followed up through 1995 (mean 5.5 years) for cardiovascular outcomes and death. The 7-year survival rate was 93% for the HRT users versus 75% for the non-users. The rate of cardiovascular death or myocardial infarction was 62% lower with HRT use (RR 0.38; 95% CI 0.19–0.79), with the conclusion that HRT use is associated with improved long-term outcomes after PTCA in postmenopausal women. In this study as well *immortal time bias* is introduced by defining use of HRT not only at the time of PTCA but also during the follow-up period. Thus initiators of HRT during this follow-up are misclassified as exposed before they started HRT use, when they should have been classified as non-users up to that point, thus leading to immortal time bias.

## METFORMIN AND CANCER

Metformin is a drug of choice for the management of type 2 diabetes mellitus.^46^ It reduces insulin resistance and improves glycemic control and can be combined safely with other anti-diabetic drugs.^47^ In 2005, an observational study using data from Tayside, Scotland, reported a significant 23% reduction in the incidence of any cancer with metformin use, thus advancing the hypothesis that metformin could lower the risk of cancer onset in patients with diabetes.^48^ This study generated great interest in metformin as an agent in cancer prevention and treatment, with many preclinical studies showing that metformin can inhibit the growth of cancer cells *in vitro* and *in vivo*.^49^–^51^

In parallel, a series of observational studies conducted in various databases generally reported similar beneficial results with metformin, thus “confirming” the findings of the 2005 study. A meta-analysis including some of these observational studies reported that their combination resulted in a highly significant 31% reduction in cancer incidence or mortality associated with metformin use (RR 0.69; 95% CI 0.61–0.79).^52^ This convergence of evidence from multiple preclinical and epidemiological studies formed the impetus to recommend the conduct of randomized controlled trials (RCTs) of metformin in cancer prevention and treatment.^53^,^54^ There are currently many trials of this issue registered in *clinicaltrials.gov.*

The many observational studies conducted to date will not be reviewed in detail here as they are the object of a separate paper.^55^ These observational studies have not only looked at whether metformin lowers cancer incidence and mortality,^56^–^59^ but also whether metformin can act as a treatment of cancer to lower cancer mortality or recurrence.^60^ However, many of these observational studies that report significant reductions in cancer incidence and mortality and better prognosis with metformin use, with spectacular reductions ranging from 20% to 90%, are affected by time-related biases such as immortal time bias.^34^,^35^,^55^ This bias is known to exaggerate downward the effect of a drug, thus making a drug appear protective when it in fact it may have no effect.

On the other hand, two recent observational studies that specifically used the proper time-dependent statistical techniques to properly classify metformin exposure found no association between metformin use and cancer incidence.^61^,^62^ The first study used the GRPD and found a rate ratio of prostate cancer incidence of 1.23 (95% CI 0.99–1.52) with metformin use. With more than 36 prescriptions the rate ratio was in fact significantly elevated at 1.40 (95% CI 1.03–1.89).^61^ The second study used the Kaiser Permanente database and found no effect of metformin on the incidence of the 10 different cancers studied, with hazard ratios ranging between 0.8 (95% CI 0.6–1.1) for melanoma to 1.3 (95% CI 1.0–1.6) for kidney/renal pelvis.^62^

## CONCLUSION

Many observational studies conducted to uncover new indications for drugs that are already on the market have been shown to have major methodological flaws leading to important biases that tend to falsely suggest that a drug is highly effective. In particular, immortal time bias appears to be a major culprit in these studies. In two of the situations that we presented, this apparent effectiveness was not confirmed in subsequent large-scale randomized controlled trials conducted to evaluate these findings. Indeed, the numerous observational studies of hormone replacement therapy (HRT), indicated for menopausal symptoms, and suggesting cardiovascular benefits, were clearly flawed; the WHI randomized trials did not confirm such benefit. Similarly, the observational studies of inhaled corticosteroid treatment, indicated for asthma but used in COPD without evidence, suggested spectacular benefits of these drugs on reducing all-cause mortality, benefits which were subsequently not corroborated by the large TORCH randomized trial. Currently, history may be repeating itself with the anti-diabetic medication metformin which has been the subject of several observational studies that reported impressive reductions in the incidence of and mortality from cancer. These spectacular “beneficial” anti-cancer effects are clearly again the result of time-related biases which tend to exaggerate the benefits observed with a drug. Yet, these observational studies form the basis for the conduct of large-scale randomized trials currently underway.

Interestingly, with such promising findings from observational studies, many animal studies are conducted to understand and describe possible mechanisms by which, for instance, metformin could prevent or slow cancer progression, or physiological explanations of the possible effects of inhaled corticosteroids on systemic inflammation in COPD and the potential benefit on mortality. Such research brings greater momentum to the new indication, eventually leading to large trials. However, it is imperative first to carry out critical assessments of the observational study methods, for which possible methodological explanations for these “spectacular” results have received little attention (see Box 1). While these biases are well-known in pharmacoepidemiology and have been described extensively in different therapeutic areas,^31^,^34^,^35^,^63^,^64^ they do not seem to have yet sufficiently penetrated different subspecialty fields such as diabetes, cancer, pulmonary medicine, etc.

Box 1. **How to Detect Immortal Time Bias During Peer Review**

If a cohort study reports extreme beneficial effects (relative risks < 0.70) for a treatment, reviewer should consider immortal time bias as an alternative explanation and answers in the affirmative to any of the following questions should raise red flags:

- Was treatment status determined during the follow-up time?
- Was the start of follow-up different for the treated and comparison groups?
- Were the treatment groups formed hierarchically, first the treated group and next the comparison group?
- Were subjects excluded on the basis of treatment initiated during follow-up?
- Did the analysis report data on patients and not person-time to reflect time-varying exposure to treatment?
- Was a time-fixed technique of data analysis used?

In summary, while observational studies are central to assess the effects of drugs, their proper design and analysis are essential to avoid bias, particularly time-related biases that tend to falsely suggest strong drug benefits. Certainly, methodologically inaccurate studies, even if their biased results are replicated in different settings and by different authors, should not be the driving force in conducting randomized trials. The scientific evidence on the potential beneficial effects in new indications of existing drugs will need to be more carefully assessed before embarking on long and expensive unsubstantiated trials.

## Abbreviations:

CABG: coronary artery bypass surgery
CHD: coronary heart disease
COPD: chronic obstructive pulmonary disease
GRPD: General Research Practice Database
HRT: hormone replacement therapy
ICS: inhaled corticosteroids
LABA: long-acting beta(2)-agonist
PTCA: percutaneous transluminal coronary angioplasty
RCT: randomized controlled trial
WHI: Women’s Health Initiative
